# CORESS is an independent charity, supported by AXA Health, the MDU and the Kirby Laing Foundation

**DOI:** 10.1308/rcsann.2023.0100

**Published:** 2024-03-01

**Authors:** H Corbett

**Affiliations:** on behalf of the CORESS Advisory Board

## Abstract

We are grateful to those who have provided the material for these reports. The online reporting form is available on the website (coress.org.uk), which also includes previous Feedback reports, and via the CORESS app. Published cases will be acknowledged by a Certificate of Contribution, which may be included in the contributor’s record of continuing professional development, or which may form part of appraisal or annual review of competence progression portfolio documentation. Trainee contributions are particularly welcome.

## Lack of communication in patient discharge

### Case 283

A 63-year-old man with diabetes, chronic stage 3 kidney disease and ischaemic heart disease was admitted with a necrotic fifth toe, cellulitis and hyperkalaemia. Surgery to amputate the toe and debride localised tissue necrosis was undertaken under regional anaesthesia within 24 hours.

The wound was reviewed the next day by the consultant, who took the dressing down on the postoperative ward round. The patient was discharged with a five-day course of antibiotics and an appointment for review in the diabetic foot clinic two weeks later. However, there was no communication with the patient about the frequency of required dressing changes. No nurses were present on the ward round and no information was given to the nurses about dressing changes at a verbal handover. Nor was there a formal handover from the inpatient nursing team to the community nurses.

The patient was readmitted eight days following the original surgery with spreading sepsis and subsequently required amputation of three other toes on the same foot.

#### Reporter’s comments

This case illustrates the poor outcomes associated with failed communication at different stages in the patient journey. While the patient was seen promptly following surgery, there was failure of the surgical team to communicate crucial management issues to the nursing team responsible for the patient’s discharge. This could have been queried at this stage but it was not and no instructions were issued to the community nurses, who form a vital part of the postoperative care team. It is the responsibility of the surgical team to ensure that adequate postoperative instructions are directed to those responsible for the patient's discharge and community care. Regular team meetings of all involved in surgical patients’ care (including surgeons, nurses, physiotherapists and occupational therapists) foster a team spirit, and may enhance communication and patient care.

#### CORESS comments

A collaborative care pathway with written protocols for patient discharge and early community nursing involvement might have reduced the risk of the adverse outcome, which arose as a result of poor communication.

## Consequences of service disruption during the COVID-19 pandemic

### Case 284

A 64-year-old man presented with a mixed arteriovenous lower leg ulcer. Duplex ultrasonography and computed tomography angiography confirmed mild deep venous incompetence and a 10cm superficial femoral artery occlusion. The patient underwent femoral artery angioplasty and placement of an uncovered stent, improving his ankle-brachial pressure indices, allowing him to be placed in four-layer graduated compression bandaging to treat the venous component of his ulcer.

Stent surveillance by duplex ultrasonography would usually have been performed at routine three-monthly intervals for the first year after stent placement. However, this was postponed because of changes in routine practice due to the COVID-19 pandemic.

The patient was seen in the vascular ‘hot clinic’ as an emergency referral four months after the intervention, at which time his leg ulcers had deteriorated to the extent that tendons were exposed and there was severe necrosis of skin on the dorsum of his foot. Ultrasonography confirmed that the stent had occluded while he had remained in compression bandaging.

The foot was deemed non-salvageable and the patient underwent below-knee amputation. He was making a good recovery from amputation, with early mobilisation, when he developed a hospital-acquired COVID-19 infection. His respiratory function deteriorated rapidly, requiring admission to the intensive care unit. He developed further thrombotic sequelae of COVID-19 and systemic inflammatory response syndrome, with digital necrosis of fingers, necessitating a prolonged stay on the intensive care unit.

#### CORESS comments

The impact that the COVID-19 pandemic has had on routine clinical services is well recognised. This case is a salutary reminder that expected clinical surveillance as part of follow-up protocols after emergency interventions should be adhered to wherever possible. Telephone follow-up clinics will not be suitable for some patients. Development of improved communication links between community services and the surgical team might have helped identify continued deterioration of this patient's presenting condition.

## Too slick by half

### Case 286

A trainee surgeon, aiming to expedite a morning day-case list by efficient management of paperwork, pre-completed the consent forms for the five patients due to undergo hernia repair. This included signing and dating the forms prior to seeing the patients. He was then called away to deal with a ward emergency and a colleague took over, seeing the patients and marking the appropriate site of surgery. Seeing that the consent forms were already signed and assuming that the patients had already been seen, the colleague merely asked the patients to sign them and marked the side indicated on the consent form.

The first patient arrived in theatre and on questioning during the pre-anaesthetic check, the anaesthetic nurse noted that the patient's symptoms were on the opposite side to that marked and indicated on the consent form. It transpired that the affected side had been listed incorrectly on the theatre list, to which the surgical trainee had referred, prior to completing the consent form.

#### Reporter’s comments

Despite the trainee's best intentions, this was an inappropriate shortcut taken to try to improve efficiency at the expense of patient safety. It is the responsibility of the operating surgeon to make sure that he or she is undertaking the correct procedure on the appropriate side and site. Examination of the lesion and then marking the side/site is a vital undertaking prior to surgery. The thorough attention of the anaesthetic nurse in this case prevented the occurrence of a ‘never event’.

#### CORESS comments

This was a classic example of the ‘Swiss cheese’ effect, where several errors lined up to contribute to a ‘near miss’. The operating surgeon should check all patients before they are anaesthetised. A formal team briefing and correctly performed World Health Organization checks should have identified this problem. The psychologist on the CORESS Advisory Board noted that there is a tendency to reaffirm what has been done before, rather than to ‘check and challenge’. The paperwork should never be completed and signed off before the clinical task is undertaken.

## Tunnelling device mishap

### Case 287

A 68-year-old man underwent an obturator bypass (Figure 1) for critical limb ischaemia in order to avoid a groin scarred by chronic infection from previous surgery. This involved retroperitoneal exposure and placement of a tunnelled prosthetic graft from the iliac artery, through the obturator foramen to the medial thigh, with distal anastomosis to the superficial femoral artery. A standard tunnelling device was used with a ‘screw-in’ blunt olive, matched to the dacron graft 8mm diameter. As the tunneller was introduced through the obturator foramen of the pelvis, from the thigh, the olive tip of the tunnelling trocar became unscrewed and disconnected from the rod of the trocar, ending up lodged and inaccessible somewhere in the deep pelvic tissues.

After numerous attempts to retrieve the 1cm long bullet-shaped olive and despite on-table imaging to confirm its position, it was deemed too potentially disruptive to attempt to extract the tip. The operation was completed using a second tunneller and the metallic olive left in situ when the incisions were closed.

Postoperatively, a full explanation with diagrams was provided to the patient by the operating surgeon. No complaint arose and at the six-month follow-up review, the graft was patent with no complications.

#### Reporter’s comments

With this particular tunnelling device, the correctly-sized olive had to be selected and screwed into the trocar rod. This was undertaken by the scrub nurse while the surgeons prepared the operative field. It is possible that this was not done correctly or that the olive was mis-threaded. Nevertheless, this should have been checked by the surgeon prior to use. In the event of the olive dislodgement, a team decision was eventually made to abandon attempts at retrieval because of the risk of causing injury. A full and honest explanation to the patient helped to defuse any potential complaint.

#### CORESS comments

Unfortunately, kit failures do occasionally occur across all surgical specialties and it is sometimes in the patient's interests to refrain from retrieving an inaccessible foreign object if it is deemed that the risk of leaving this in place is significantly less than further, potentially injurious, surgical exploration. Key points related to this case are the importance of checking all surgical equipment before introducing this into the patient. In the event of such an incident, there is a duty of candour to provide a full and frank explanation to the patient. The patient should also be warned of implications of retained metallic objects with respect to potential future magnetic resonance imaging.

**Figure 1:**
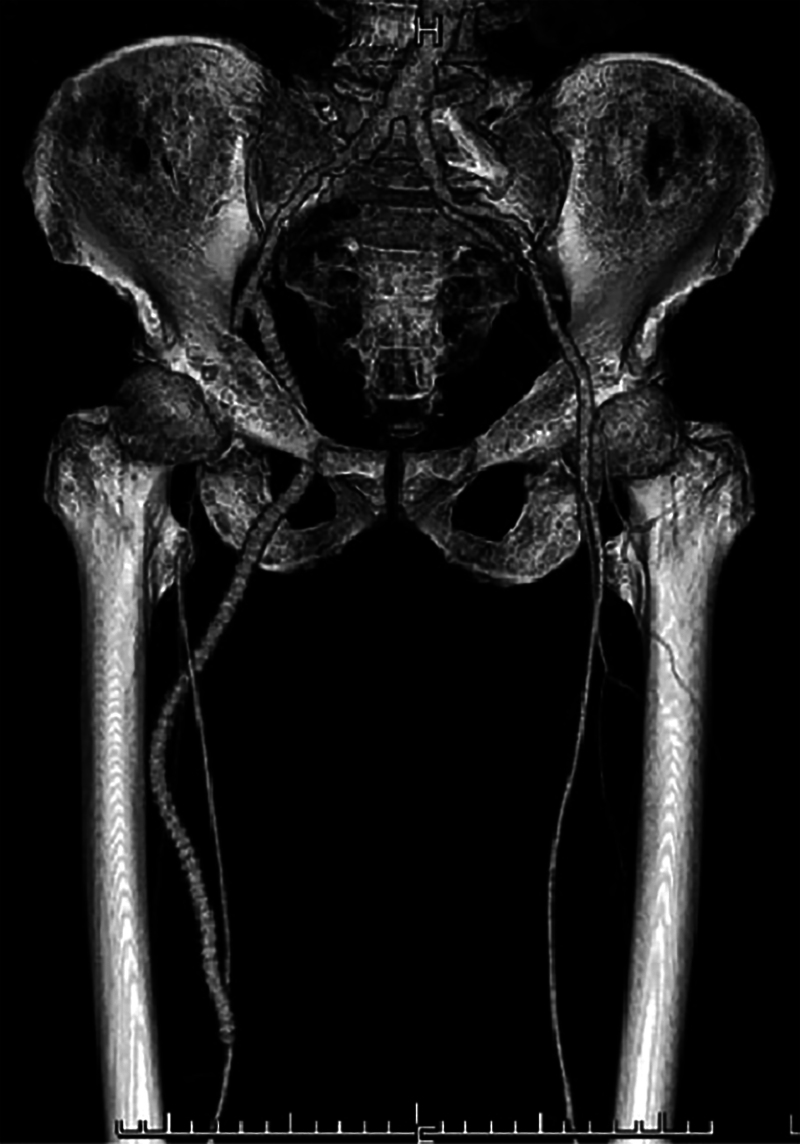
Computed tomography angiogram of right obturator bypass

## Retained tip of vein hook

### Case 288

The 38-year-old wife of a local general practitioner underwent bilateral radiofrequency ablation of incompetent varicose great saphenous veins, with concomitant phlebectomies, at a local private hospital. The procedure was carried out under general anaesthesia at her request. Phlebectomies were undertaken via small stab incisions in skin creases using a size 1 (larger) Oesch-style vein hook.

During the phlebectomies on the second limb, while removing a large anterior thigh vein varicosity, the vein hook (Figure 2) snapped at approximately 1cm from its tip, leaving the tip embedded in the thigh tissues. Attempts to locate the hook tip with a fine arterial clip were unsuccessful and despite undertaking image intensification, using crossed 21g hypodermic needles to triangulate the hook's position, it proved impossible to remove the hook tip without potentially significantly enlarging the incision. The decision was taken to complete the procedure without retrieving the hook tip. This was done without further incident.

An explanation was provided to the patient, who made a good cosmetic recovery.

#### Reporter’s comments

On completion of the operation, the set of vein hooks was inspected, and it became apparent that all had been bent through usage and probably then re-bent into shape, representing wear and tear of usage. The fracture of the hook tip had likely occurred as a result of metal fatigue rather than the use of inadvertent force.

#### CORESS comments

As in case 287 (above), a careful check of the equipment prior to use may have revealed a potential problem. Where kit is obviously worn, this should be withdrawn from use and replaced as necessary.

**Figure 1:**
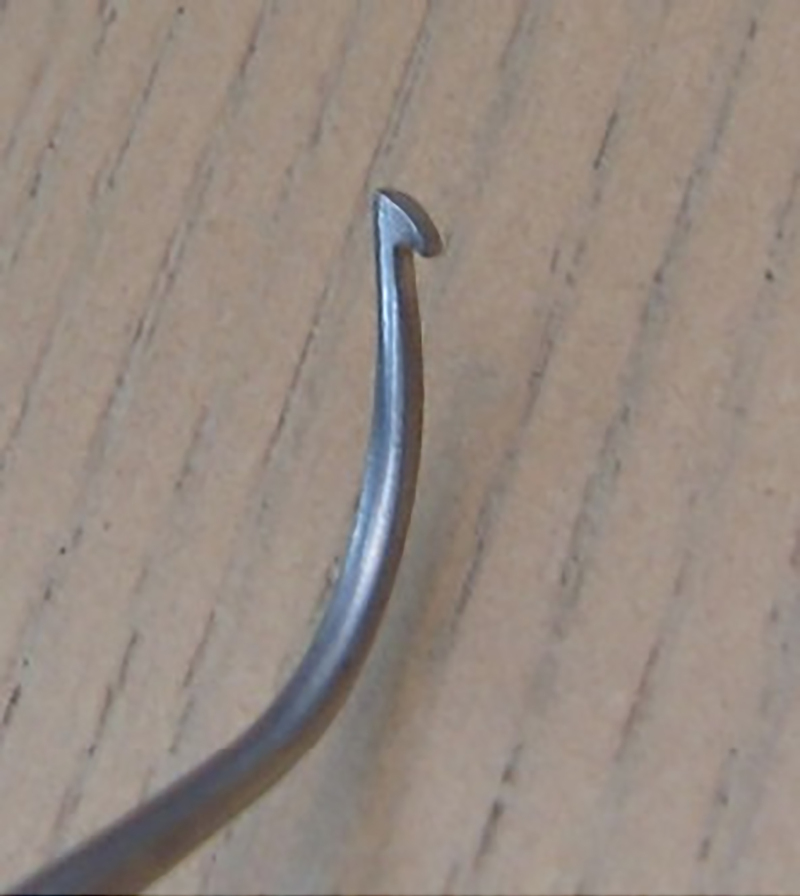
Vein hook

## Missed anal carcinoma

### Case 289

A 62-year-old woman was referred to the colorectal team with a generic letter from her general practitioner describing altered bowel habit, occasional rectal bleeding and “nasty piles”. She was booked for fast track flexible sigmoidoscopy before being seen, with a view to an outpatient appointment after the test.

A flexible sigmoidoscopy was performed by an experienced nurse practitioner and reported as normal. As a result, a routine outpatient appointment was made, which the patient attended 16 weeks after the test. At the outpatient clinic, it became evident that the “nasty piles” was an anal carcinoma.

#### Reporter’s comments

This case demonstrates the potential danger of a fast track policy in which the patient may not have been seen by a clinician with colorectal experience before the investigation. The description of “nasty piles” should have flagged up the possibility of anal or perianal pathology.

#### CORESS comments

This case raises the question as to whether the patient was examined thoroughly prior to referral to the fast track colorectal clinic. Anal examination should be undertaken before any colorectal endoscopy but if the endoscopist is uncertain of the clinical implications of abnormal appearances, a second opinion should be sought.

## Missed sepsis following laparoscopic cholecystectomy

### Case 290

A patient was readmitted for pain control five days after a difficult elective laparoscopic cholecystectomy. Ultrasonography was difficult (because of patient habitus) but unremarkable. On the following morning, the patient still had a tender abdomen and guarding but no rebound, with normal bowel sounds. Blood pressure and pulse were normal. Blood tests revealed an inflammatory response and after consultant review, the plan was for supportive therapy and repeat assessment over the weekend. The patient was handed over to the night on-call team for review.

On the following morning, a Saturday, the night registrar noted that the patient was not on the list for ward review (in our hospital, inpatients are placed on a different list from post-take patients and are reviewed by a separate surgical team) and the F1 doctor was informed. The F1 doctor did not include the patient on his list and so the patient was not reviewed subsequently on that day by the locum registrar who was covering the wards. The ward nurses responsible for the patient did not alert the surgical team to the fact that she had not been seen.

On the Sunday morning, the night on-call registrar (who knew the patient) reviewed all the blood tests from Saturday and noted a soaring inflammatory response. The surgical team went back to review the patient and found her septic, and now, with frank peritonitis. The patient underwent urgent surgical exploration, during which a subhepatic collection of old blood, bile and fibrin was washed out, and a drain placed. The patient eventually made a good recovery.

#### CORESS comments

As with many cases, a number of separate factors lined up to produce the adverse incident described here. The key underlying problem was poor communication between the different teams of staff responsible for the patient’s care. The fact that sick inpatients and post-take patients were on separate lists for review reflected a problem with the system. The F1 doctor forgot to include the patient on a list for review, the locum may not have been aware of hospital procedures and the nursing staff did not remind the on-call team that the patient needed review.

The Association of Surgeons in Training member of the CORESS Advisory Board commented that this was a ‘failure to rescue’ and introduced the board to the useful metric: ‘Recognise, relay, react’.^1^ It was noted that existence of an early warning system or escalation protocols might have prompted earlier review of the patient.
